# Recent progress and perspectives on physiological and molecular mechanisms underlying cold tolerance of tea plants

**DOI:** 10.3389/fpls.2023.1145609

**Published:** 2023-02-14

**Authors:** Yanli Wang, Lidia Samarina, Ali Inayat Mallano, Wei Tong, Enhua Xia

**Affiliations:** ^1^ State Key Laboratory of Tea Plant Biology and Utilization, Anhui Agricultural University, Hefei, China; ^2^ Federal Research Centre the Subtropical Scientific Centre, The Russian Academy of Sciences, Sochi, Russia

**Keywords:** Camellia sinensis, tea plant, cold stress, molecular mechanism, review

## Abstract

Tea is one of the most consumed and widely planted beverage plant worldwide, which contains many important economic, healthy, and cultural values. Low temperature inflicts serious damage to tea yields and quality. To cope with cold stress, tea plants have evolved a cascade of physiological and molecular mechanisms to rescue the metabolic disorders in plant cells caused by the cold stress; this includes physiological, biochemical changes and molecular regulation of genes and associated pathways. Understanding the physiological and molecular mechanisms underlying how tea plants perceive and respond to cold stress is of great significance to breed new varieties with improved quality and stress resistance. In this review, we summarized the putative cold signal sensors and molecular regulation of the CBF cascade pathway in cold acclimation. We also broadly reviewed the functions and potential regulation networks of 128 cold-responsive gene families of tea plants reported in the literature, including those particularly regulated by light, phytohormone, and glycometabolism. We discussed exogenous treatments, including ABA, MeJA, melatonin, GABA, spermidine and airborne nerolidol that have been reported as effective ways to improve cold resistance in tea plants. We also present perspectives and possible challenges for functional genomic studies on cold tolerance of tea plants in the future.

## Introduction

Tea is one of the most popular nonalcoholic beverages favored by worldwide consumers, and represents a valuable economic, healthy, and cultural values. It is an evergreen perennial plant that belongs to the genus *Camellia*, which contains over 200 species (Banerjee, 1992; Cetinbas-Genc et al., 2020; Wu et al., 2022). Tea plants are often grown in tropical and subtropical regions ranging from 49° N in Ukraine to 33° S in South Africa, making them susceptible to cold weather (Carr, 2008; Tuov and Ryndin, 2011; Hao et al., 2018). In general, soils with a pH range of 4.5-6.5, high humidity levels, and the temperature of 21-29°C are the best conditions for tea plant cultivation. The continuing deterioration of the environment and particularly cold stress have seriously threatened the sustainable development of global tea industry (Carr, 2008; Yadav, 2010). Tea plants have a long lifespan, which means that their physiology has to be able to adapt to different temperatures in order to survive in extreme environments. Therefore, it is important to elucidate the molecular mechanisms that are implicated in response to cold tolerance in tea plants.

Low temperature is one of the most pivotal environmental factors that affects tea growth, yields, and quality. Under cold stress, tea plants experience extensive physiological and biochemical changes, including the alternations of cell membrane fluidity and protein activity, as well as the release of many bioactivities such as reactive oxygen species (ROS) and malonaldehyde (Theocharis et al., 2012). Likewise, during cold acclimation of tea plants, protective osmoregulation such as soluble sugars, amino acids (like proline) and some amines (like polyamines) all were significantly accumulated (Ding et al., 2020; Li et al., 2020; Wang et al., 2020). The cold-tolerant properties of tea plants could be attributable to stronger palisade parenchyma and reduced stomata density compared to sensitive varieties (Samarina et al., 2020). Since tea plants are sessile and unable to escape the adverse environment, they have to develop relevant responsive mechanisms to adjust to cold stress. In plants, the cold stress is perceived by cold sensors and transduced by cold signaling, which involves a series of kinases, light receptors, calcium channels and NO signals, and is closely correlated to the core *ICE (INDUCER OF C-REPEAT BINDING FACTOR) -CBF (C-REPEAT BINDING FACTOR 1) -COR (COLD-RESPONSIVE GENE)* pathway or CBF-independent way. In particular, the discovery of cold sensor of rice (COLD1) and thermosensors of rice (TT3) and Arabidopsis (ELF3) have greatly enhanced our understanding of the plant temperature adaptability (Ma et al., 2015; Jung et al., 2020; Zhang et al., 2022a). Plethora of recent studies have revealed the important roles of transcriptional, epigenetic, and post-transcriptional regulations in cold signaling (Fowler and Thomashow, 2002; Barrero-Gil and Salinas, 2013; Mann and Jensen, 2003).

One of the effective ways to improve the cold resistance in tea plant is to identify the key genes responsible for cold tolerance of tea plant and then use transgenic or cross-breeding methods to breed new germplasms with high cold tolerance. Consequently, investigation of the cold response mechanisms in tea plant is fundamental for molecular breeding. Here, we summarized the research progress of tea plants in cold response, including molecular, physiological and biochemical responses to cold stress, sensing and signal transduction, cold-responsive gene identification, and their transcriptional regulation and post-transcriptional modification during cold stress. We also propose that application of exogenous protective substances such as ABA, MeJA (Methyl Jasmonate), melatonin, GABA, spermidine, and airborne nerolidol is expected to effectively improve the cold resistance of tea plants. The key challenges and future prospects are also discussed. Despite the current findings on molecular mechanism of cold tolerance of tea plants are rather limited, the development of omics would extend our understanding for sophisticated network of low-temperature regulation. Our goal is to offer potential future avenues for the development of cold-responsive systems that could serve as a resource for woody plant breeding research.

## Physiological changes of tea plants during cold stress

Low temperature inflicts irreversible disorders to plant physiology, posing threats to the survival and sustainable development of plants. Cold stress can lead to leaf senescence, seedling death, pollen abortion, bud dormancy, pollen tube abnormality, and especially damaging the tea shoots and inhibiting their growth (Cetinbas-Genc et al., 2020; Zheng et al., 2016; Hao et al., 2017; Song et al., 2017; Xiao et al., 2018). Cold stress caused by extreme temperature fluctuations commonly includes chilling stress, frost stress, and freezing stress. In general, chilling stress occurs at the temperature of 0-15°C, while most frost damage emerges at a clear night, wherein radiation freezing air occupies the area. Cold stress usually solidifies the plasma membrane elasticity and phospholipids fluidity, and affects the channels and increases permeability of plants. The disturbed permeability will then lead to electrolyte leakage and enzyme inactivation for the lack of optimal temperature and pH (Lukatkin et al., 2012). Unlike chilling and frost stress, freezing stress often occurs when air temperature is below 0°C, which results in the formation of an ice crystal in plant tissues (Yadav, 2010; Shi et al., 2018). Compared to chilling stress, freezing stress is usually lethal. It forces the intracellular water into extracellular ice, and damages the integrity of membrane, accompanied by the disruption of cellular compartmentalization and denaturation of membrane proteins (Wisniewski et al., 2004).

**Figure 1:**
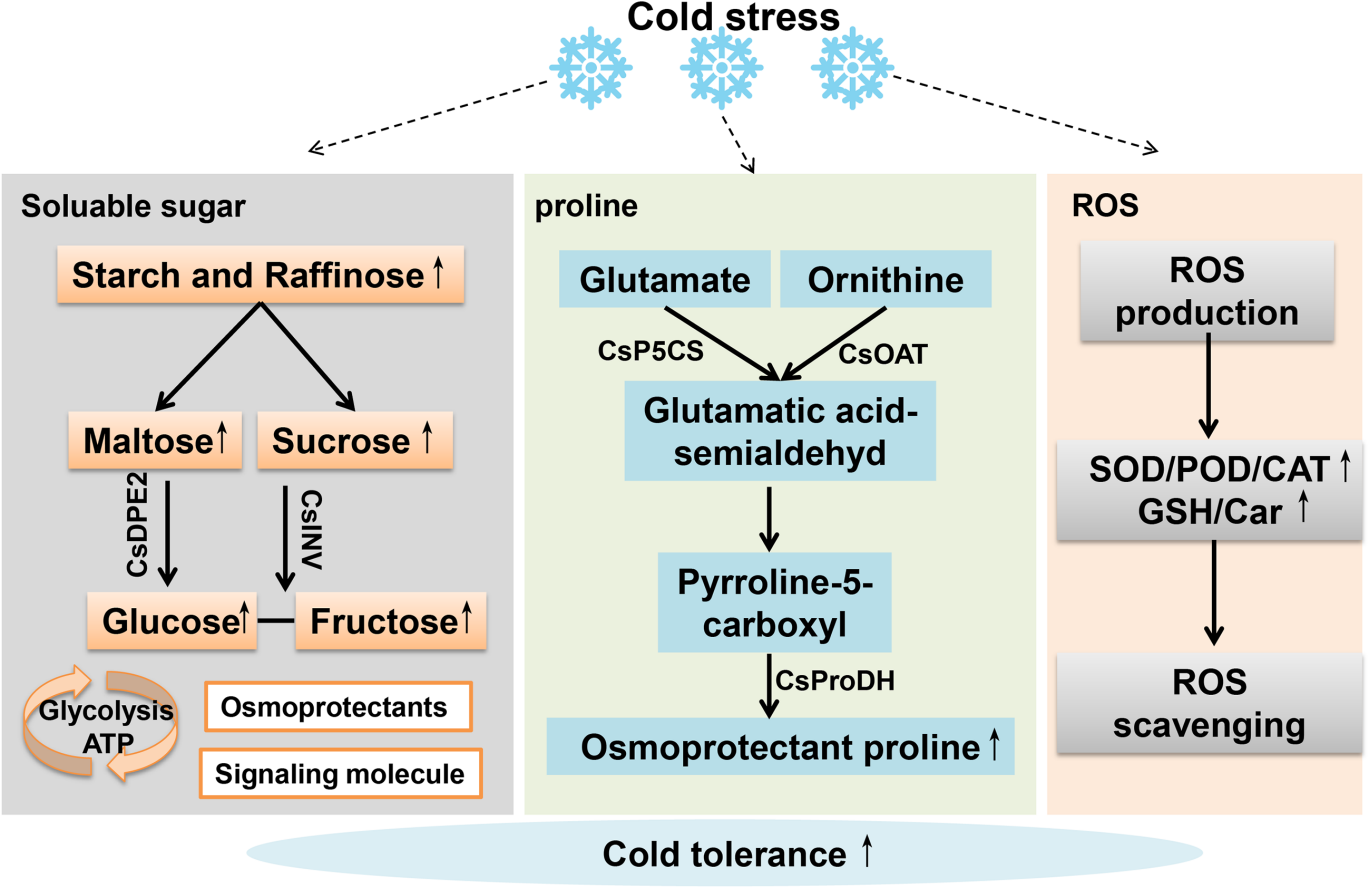
The alternation of some metabolisms of tea plants during cold stress.

The stress response of plants is a complex and dynamic process. Cumulative evidences have shown that hundreds of metabolic processes of tea plants altered under cold stress (Shen et al., 2015). The metabolism of soluble sugars, proline and reactive oxygen species (ROS) are among the most pronounced changes. Soluble sugars level is highly sensitive to cold stress. As is shown in **Figure 1**, cold stress leads to the conversion of polysaccharide to disaccharide, which then yields glucose and fructose to accelerate the accumulation of soluble sugars. The sugars then act as osmoprotectants interacting with lipid bilayer and stabling protein folding to confer plants with cold tolerance (Ma et al., 2009). Besides, the soluble sugars were also found to function as a signaling molecule to regulate the crosstalk of hormones to activate the expression of cold responsive genes in response to cold stress of plants (Couée et al., 2006; Rolland et al., 2006; Janská et al., 2010). Similarly, cold and frost stress can significantly elevate the protein levels (by 3-4 folds), and contents of proline and cations (potassium, calcium and magnesium), which will serve as a common compatible osmolytes to decrease ice point and molecular chaperone to scavenge reactive oxygen species to rescue the cold damage in plants (**Figure 1**, Yoshiba et al., 1997; Ghosh et al., 2022).

## Mechanisms of sensing and signal transduction of tea plants during cold stress

### Sensing of cold signals in tea plants

**Figure 2:**
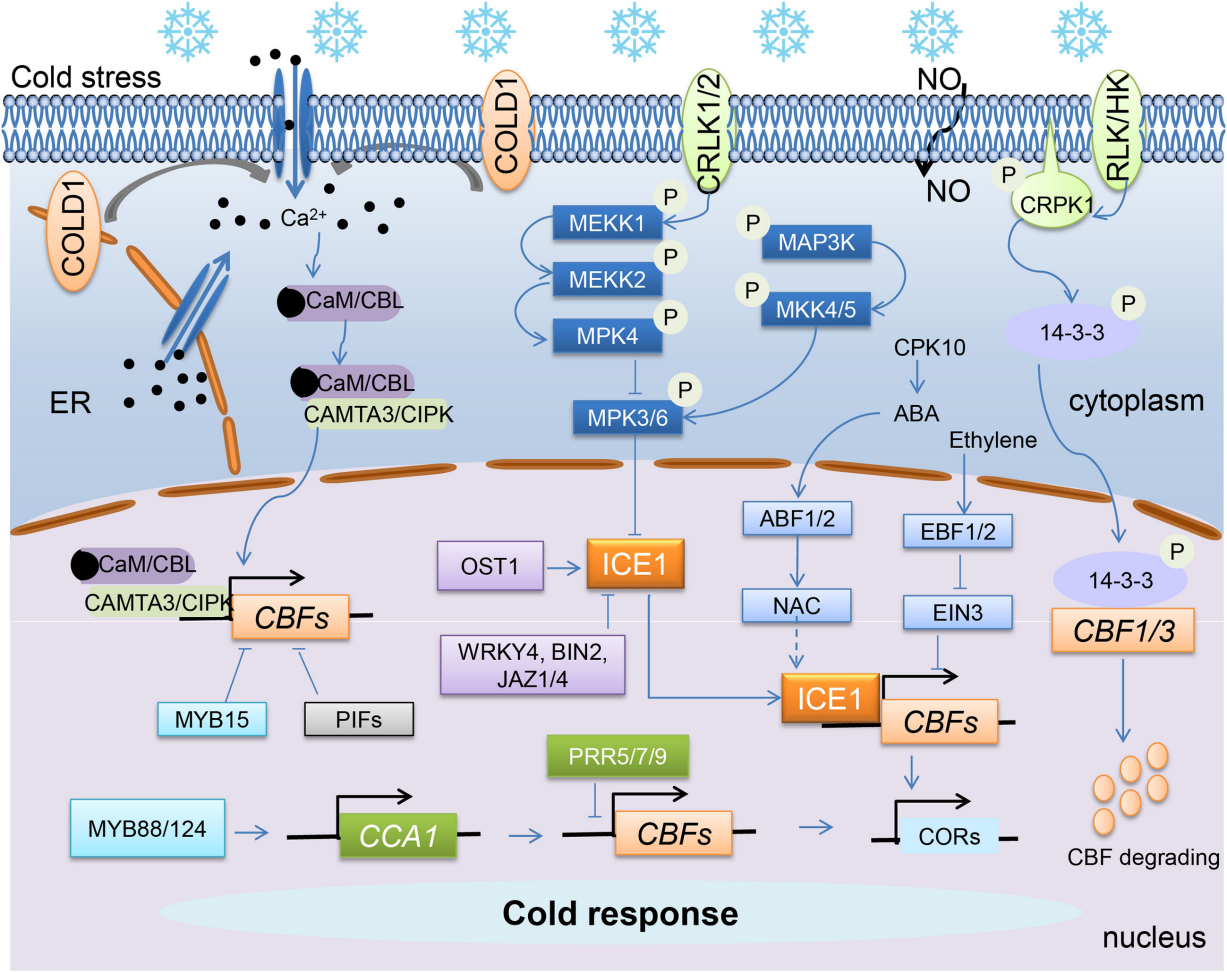
Cold sensors and signal transduction in tea and other plant species.

Being sessile organisms, plants have evolved relative elaborate mechanism to sense and cope with the ever-changing temperature. Cold signals are sensed by receptors through the cell membranes, which then participate in the regulation of intracellular signaling networks or cell-cell communication (Ahuja et al., 2010; Norman et al., 2011; Jogaiah et al., 2013). Much efforts have been made to discover the cold sensors (**Figure 2**). The CHILLING TOLERANCE DIVERGENCE 1 (COLD1) is one of the currently identified cold sensors in rice, which could mediate the chilling tolerance of rice by regulating calcium channels and *OsCBF1* gene expression (Ma et al., 2015). It deserved to further investigate whether the function of *COLD1* gene is conservative in tea or other plant species. In addition to COLD1, the receptor-like kinases (RLKs) and histidine kinases (HKs) were also able to sense environmental signals. During cold acclimation of tea plants, almost all *CsRLK* and *CsHK genes* were particularly found to be up-regulated, suggesting their crucial roles for cold tolerance in tea plants (Wang et al., 2013). RLK members constitute the largest gene family of plant membrane signaling proteins, while HKs are the most abundant and diverse membrane receptors. Both of them are potential cold sensors in plants (Osakabe et al., 2013). They usually regulate the expression of many cold-inducible genes through abscisic acid signaling pathway and/or calcium/calmodulin signaling or other manners under cold stimuli (Murata and Los, 2006; Yang et al., 2010; Xu et al., 2020).

Besides, previous studies demonstrated that the light receptors were also closely related to cold response of bacteria and plants. They reported that cold-stress signaling pathways showed to be closely associated with the light perception and circadian clock, but it remains unknown how plants sense and transmit stress signals to regulate gene expression (Chen et al., 2004; Gould et al., 2013; Estravis-Barcala et al., 2020; Kidokoro et al., 2021; Kidokoro et al., 2022). An activation of the phytochromes under long day conditions triggers PIF7 (phytochrome interacting factor 7) to interact with the circadian oscillator TOC1, subsequently bind to a G-box sequence (CAGTG) in the CBF promoter, and thereby downregulates CBF expression (Wisniewski et al., 2014). The blue light receptor phytotropin perceives cold signals in liverwort *Marchantia polymorpha* at its photoactivated state (Fujii et al., 2017). In Arabidopsis, phytochrome B (phyB) photoreceptor integrates light and ambient temperature by reversible photoconversion between active Pfr (far-red) state and the inactive Pr (red) light-absorbing state (Legris et al., 2016). The physical interaction of phyB and CBF1 abrogated the interaction of phyB and PIF3/4 to promote the photomorphogenesis at 22/17°C in the light (Lu et al., 2020) (Dong et al., 2020) Jung et al., 2016; Legris et al., 2016). In contrast, the cold-induced *CBFs* stabilized the phyB thermosensor to enhance plant cold tolerance at 4°C (Jiang et al., 2020). In plants, phytochrome signaling pathway related gene *AtFHY3/FAR1* (*FAR-RED ELONGATED HYPOCOTYL 3/FAR-RED-IMPAIREDRESPONSE 1*) not only modulates the phyA activity by directly activating the expression of *FHY1/FHL* but also positively regulates cold response through regulating JA (jasmonic acid) signaling pathway (Liu et al., 2019; Dai et al., 2022). Unfortunately, little research has been done on the interaction of low temperature and light signals in tea plants, with the exception of the expression analysis of *CsFHY3*/*CsFAR1*. Almost all of the *CsFHY3/CsFAR1* family members were down-regulated under cold stress of tea plants, suggesting their negative roles for regulating cold tolerance in tea plants (Liu et al., 2021).

An increased expression in a set of genes related to red-light perception (*GRAVITROPIC IN THE LIGHT*), blue and UV-light perception (*CRYPTOCHROME 1*, *EARLY LIGHT-INDUCABLE PROTEIN 1*, and *UVB-RESISTANCE 8*), chloroplasts relocation (*CHLOROPLAST UNUSUAL POSITIONING 1*), and regulation of chlorophyll biosynthesis (*Cs213* putative cold-inducible protein), stomatal movement (*PHOSPHOGLUCOMUTASE*), PSII associated light-harvesting complex II catabolic process (*FILAMENTATION TEMPERATURE-SENSITIVE H*) were also observed in tea plant under the long term cold stress. Interestingly, among all upregulated DEGs (differential expressed genes), the highest expression level was observed in *ELIP1* (*EARLY LIGHT-INDUCIBLE PROTEIN 1*) which was upregulated 150-2000 folds above control under long-term cold stress in tea leaves. ELIPs are located in thylakoid membranes and are known to protect photosynthetic machinery from various environmental stresses in higher plants and have been reported to participate in the phytochrome signaling pathway (Rizza et al., 2011). Additionally, it was reported that the induction of *ELIP1*/2 expression is mediated *via CRY1* (*CRYPTOCHROME 1*) in a blue light intensity-dependent manner (Kleine et al., 2007; Yang et al., 2017). During exposure to high irradiance, *cry1 Arabidopsis* mutants displayed inhibition of anthocianidin and flavonoids biosynthesis genes and phenylpropanoid genes, peroxidase genes, *GST* and *ERD9* genes which are the components of various stress responses (Kleine et al., 2007). In our RNAseq study, the expression of *CRY1* was also significantly increased under the 14 days of chilling stress and 3-day freezing temperature of tea plants. According to the previous data, *CRY1* participates in the high temperature response in plants (Ma et al., 2016), suggesting that the mechanisms of temperature compensation might in principle be linked to the mechanisms of light perception (Gould et al., 2013), however the authors did not observe an accumulation of CRY transcripts under short-term cold stress in *Arabidopsis*.

Additionally, several new identified DEGs were upregulated under the long-term cold stress of tea plants which seems to be related to light sensing pathways. Among them, EID1 (EMPFINDLICHER IM DUNKELROTEN LICHT 1) -like F-box protein 3 is an F-box protein that related to red-light perception and functions as a negative regulator in phytochrome A (phyA)-specific light signaling. F-box proteins are components of SCF ubiquitin ligase complexes that target proteins for degradation in the proteasome, regulates photomorphogenesis and flowering in *Arabidopsis* (Marrocco et al., 2006). Similar to the previous finding (Ohta and Takaiwa, 2014), we identified *DNAJ11* and *DNAJ ERDJ3B-like* in tea plant which encode co-chaperone components, stimulate Hsp70 ATPase activity, which is responsible for stabilizing the interaction of Hsp70 with client proteins. Knockout of these genes in *Arabidopsis thaliana* caused a decrease in photosynthetic efficiency, destabilization of PSII complexes and loss of control for balancing the redox reactions in chloroplasts (Chen et al., 2010). Also, FTIP 1/3 (*FLOWERING LOCUS T-INTERACTING PROTEIN 1/3*) was upregulated in tea plant under the long term cold stress. This gene is an essential regulator of FT encoding florigen and the regulator of photoperiodic control of flowering in plants. Loss of function of *FTIP1* exhibits late flowering under long days, which is partly due to the compromised FT movement to the shoot apex (Liu et al., 2012). To summarise our results on tea RNAseq data, it can be suggested that the long term overlapping stress responses include the activation of several important genes of photo-perception which probably activate the phenylpropanoid pathway leading to the cell wall remodeling.

### Messenger molecules involved in cold signal transduction

The cold signals perceived by plant cell-surface could be transmitted to the other cell compartments, such as the nucleus, where the expressions of many cold-responsive genes are activated (Zhu, 2016). An example of such mechanisms include Calcium (Ca^2+^) and IP_3_ (inositol-1, 4, 5-triphosphate), which are two ubiquitous secondary messengers and play a crucial role in eliciting downstream cold-responsive signaling pathways (Knight et al., 1996; Virdi et al., 2015; Paknejad and Hite, 2018). Cold stress causes an activation of the IP_3_-gated calcium channels, which then resulted in rapid induction of cytosolic calcium levels (Ca^2+^ spark) and subsequent upregulation of *COR* genes (Orvar et al., 2000; Sangwan et al., 2001). After sensing and interacting with Ca^2+^, calmodulin (CaM) undergoes conformational changes to activate CaM-binding transcription activator (CAMTA) factors to response to cold stress. The double mutations of *camta1* and *camta3* impaired cold tolerance of plants compared to wild type, suggesting their significant roles in the cold response. Many studies have also shown that the expression of *CAMTA3*/5 is significantly upregulated under cold stress, in which CAMTA3 further binds the *CBF2* promoter to activate the expression of *DREB1B* and *DREB1C* genes. This establishes a link between calcium signals and cold acclimation in plants (Doherty et al., 2009; Eckardt, 2009; Kidokoro et al., 2017). Additionally, cold stress can stimulate the Ca^2+^ sensors calcineurin B-like protein (CBL), to interact with CBL-interacting serine/threonine-protein kinases (CIPKs), and ultimately increase the autophosphorylation and phosphorylation activity of CIPKs. The activation of CIPKs eventually resulted in an upregulation of *CBFs* genes to respond the cold stress in plants (Zhang et al., 2019). Overall, the above evidences indicated that Ca^2+^-CaM/CBL-CAMTA3/CIPK complex enables sensing and transduction of cold signaling of plants in response to cold stress through *CBF*-dependent pathways, despite their functions in tea plants need further investigations.

Under the long-term cold stress, we also observed the elevated expression of several genes, related to Ca^2+^-dependent signaling and protein phosphorylation in tea leaves. Among them, CNX1 (Calnexin) and calreticulin-like (CRT) which were reported to bind proteins on endoplasmic reticulum acting as molecular chaperones (Liu et al., 2017; Joshi et al., 2019); also, CIPK12 and CIPK6 which were reported to bind CBLs regulating Ca^2+^-signal response (Sardar et al., 2017; Czolpinska and Rurek, 2018; Bai et al., 2022). Additionally, several genes encoding the important components of membrane trafficking system and related to Ca^2+^-signaling were upregulated in tea plant under the long term cold stress, such as *STRAP* (*SERINE-THREONINE KINASE RECEPTOR-ASSOCIATED PROTEIN*)*, SAPK3* (*SERINE/THREONINE-PROTEIN KINASE 3*)*, leucine-rich repeat receptor-like protein kinase PXY1* (*PHLOEM INTERCALATED WITH XYLEM-LIKE 1*)*, INPP5A2 type I inositol polyphosphate 5-phosphatase 2, glycine-rich protein A3-like GRP* (*GLUTAMINE-RICH PROTEIN*), indicating their important roles in response to long-term cold stress in tea plant. Similarly, in *Populus*, the *calcium-dependent protein kinase 10* (*CPK10*) is upregulated under drought and frost and activates both drought- and frost-responsive genes to induce stress tolerance (Chen et al., 2013). In apple DEGs encoding protein phosphatases and serine/threonine-protein kinases were upregulated in response to different abiotic stresses (Li et al., 2019).

In addition, the *protein phosphatase 2C* (*PP2C*) was upregulated in tea plant under the long-term cold stress. PP2C are the key players in plant signal transduction processes, acting as the central components in ABA signal transduction and negative regulators of mitogen-activated protein kinase (MAPK) pathway (Rodriguez, 1998). Also, probable *translation initiation factor eIF-2B* (*tif224*) is increasingly expressed under 14-day-chilling and 3-day freezing stress in tea plant. This gene encodes a protein which is activated through phosphorylation by stress-sensing kinases, and leads to reduced levels of ternary complex required for initiation of mRNA translation under stress conditions (Wang et al., 2021). Our results confirmed that activation of Ca^2+^-signaling cascades is relevant to not only the short-term cold response but also for the long-term chilling and freezing-responses in tea plant.

Unlike Ca^2+^ and IP_3_, the nitric oxide (NO), a gaseous signaling molecule in plants has gained much attention for its roles in cold tolerance. Cold acclimation induced a high expression level of *nitratereductase 1* (*NIA1*) and stimulated the nitrate reductase (NR) activity, which was attributed to NR-dependent NO synthesis and eventually resulted in freezing response of plants (Zhao et al., 2009). Previous studies have suggested that NO could greatly induce the expression level of the *S-adenosylmethionine synthetase* (*MfSAMS*) gene in leaves of *Medicago sativa* subsp*. falcata*. Overexpression of *MfSAMS* in plant significantly improved cold tolerance of transgenic plants *via* up-regulating polyamine synthesis and oxidation (Guo et al., 2014). *In vitro* application of 0.02 mM NO could dramatically reduce the chilling injury index in tomato fruit by up-regulating the expression of *LeCBF1*, whereas NO inhibitors cause severe chilling injury (Zhao et al., 2011). Similarly, supplementing 500 uM NO *in vitro* caused the tea plants to significantly accumulate osmoregulation substances (e.g., soluble protein, soluble sugar, and proline) and activate superoxide dismutase and catalase. The expression levels of *CsICE1* and *CsCBF1* genes were up-regulated by exogenous NO, thereby alleviating the damage of cold to tea leaves under cold stress (Pan et al., 2016; Wang et al., 2021). However, it is unknown whether NO regulates CBF-dependent or -independent pathways in response to cold stress in tea plants, which needs further investigation.

## Regulatory mechanisms of cold tolerance of tea plants

### ICE-CBF-COR pathway in cold response of tea plants

Many plants have evolved sophisticated cold response mechanisms to survive in cold stress during long-term evolution (Thomashow, 1999; Kalberer et al., 2006). It is commonly acknowledged that the *ICE1-CBF-COR* transcriptional cascade is one of the key cold signaling pathways, which is highly conserved in tea and other flowering plants. Plant genomes contained two copies of the *ICE* gene. The ICE1 protein was found particularly abundant in the MYC-binding sites (CANNTG) of the *CBF* promoter (Chinnusamy et al., 2003). A handful of studies have shown that *ice1* mutation blocked the expression of the *CBF3* gene, whereas overexpression of *ICE1* significantly increased the expression of *CBF3* in transgenic plants (Tang et al., 2020). Besides, the ICE2 is considered a redundant duplicate of ICE1, which performs similar activities in plants in terms of cold responsiveness (Fursova et al., 2009; Kim et al., 2015). In plants, ICE typically regulates a large number of downstream genes in response to cold stress, of which CBFs serve as one of the most important targets (Vogel et al., 2005; Wang et al., 2012). CBFs act as the on/off switches of cold response. Their expression levels could be rapidly induced within 15 minutes under cold treatments, affecting the expression of over 4000 putative downstream target genes such as *COR15a, COR47*, and *COR6.6* (Gilmour et al., 1998; Seki et al., 2001; Maruyama et al., 2004; Maruyama et al., 2009; Park et al., 2015; Zhao et al., 2016; Shi et al., 2018). In *Arabidopsis*, the CBF gene family is composed of three tandem genes located on chromosome IV and exhibits consistent expression patterns in response to cold stress. *CBF1,3*-overexpressed *Arabidopsis* plant had increased freezing tolerance, while *cbfs* mutants were vulnerable to freezing stress (Jaglo-Ottosen et al., 1998; Liu et al., 1998; Medina et al., 1999). Unlike *Arabidopsis*, five *CsCBF* members have been identified in tea plants (Wang et al., 2019). In another investigation, six *CsCBF* genes were predicted (Hu et al., 2020). Interestingly, all the *CsCBFs* were strongly upregulated under cold stress, with the exception of *CsCBF3* (TEA010806). Overexpression of *CsCBF1* (GenBank EU563238), *CsCBF2* (KC702795), *CsCBF3* (EU857638), and *CsCBF5* (TPIA CSS001387) in *Arabidopsis* and *Nicotiana* displayed an enhanced cold tolerance, with increased photosynthesis ability, high level of proline, sugar and ROS content, but reduced malondialdehyde under cold stress compared to wild type (Chang et al., 2012; Yin et al., 2016; Zhou et al., 2022a; Zhang et al., 2022b). CBF proteins can recognize C-repeat/dehydration- responsive motif (CCGAC, CRT/DRE) in the promoters of a subset of *COR* genes and activate the expression of *COR* genes. It was predicted that a total of 685 potential *COR* genes were regulated by *CsCBF* in tea plants, including circadian rhythms and hormone signaling genes (Wang et al., 2019). Although the overexpression of *COR15A* and *CsCOR1* has no discernible effect on the survival of plants under cold stress, most *COR* genes greatly contribute to cold tolerance in plants (Jaglo-Ottosen et al., 1998; Li et al., 2010b). For example, the expression of *RD29A* was induced by CBF3, thereby improving the survival of frozen plants (Liu et al., 1998). Further, CBF1 occupies the clock genes *LUX* promoter. *LUX* is required for plants to survive in freezing stress (Chow et al., 2014). Interestingly, many previous studies also suggested that alternative splicing event is likely to drive the regulation complexity of *CsCOR* during cold acclimation (Li et al., 2020). It is possible that the alternative splicing of *CsCOR* plays an important role in cold acclimation of tea plants.

### Transcriptional and post-transcriptional regulation of ICE-CBF-COR genes

It is well recognized that both transcriptional regulation and post-translational modifications play important role in regulating the *CBF* cascade pathway. According to recent studies, the expression of *CBFs* and the stability and transcriptional activity of ICE1 are very important for cold tolerance. The *ICE1/2* are constitutively expressed (Tang et al., 2020). Previous studies have suggested that the phosphorylation, ubiquitination and sumoylation of ICE1 greatly regulates *CBF* expression by changing its own protein stability and transcriptional activity (**Figure 2**, Shi et al., 2018; Ding et al., 2020). Indeed, ICE1 is ubiquitinated and degraded by the high expression of osmotically responsive gene 1 (HOS1, E3 ubiquitin ligase), leading to the instability of ICE1 protein and low expression of *CBF* (Dong et al., 2006; Park et al., 2011). Whereas cold-activated SUMO E3 ligase SIZ1 (SAP and Miz) -mediated sumoylation of ICE1 increases its stability, positively regulating the cold tolerance in plants (Miura et al., 2007). In addition, three protein kinases also mediated the post-translational modification of ICE1. Low temperature induces the open stomata 1 (OST1) kinase activity, which then interacts with ICE1 and HOS1, improving the ICE1 activity and suppressing HOS1-mediated ICE1 degradation (Ding et al., 2018). By contrast, the other two protein kinases Brassinosteroid-insensitive 2 (BIN2) and mitogen-activated protein kinase 3/6 (MPK3/6) interacts with, and phosphorylate ICE1, which promoted the degradation of ICE1 (Li et al., 2017a; Ye et al., 2019). Moreover, MPK6 attenuated the inhibitory effect of MYB15 on *CBF* expression to enhance freezing tolerance in *Arabidopsis* (Agarwal et al., 2006; Kim et al., 2017). Mechanistically, jasmonate-zim-domain protein 1/4 (JAZ1/4) can also inhibit the CBF translational activity by interacting with ICE1/2 in *Arabidopsis* (Hu et al., 2013a). The most recent study in tea plants found that CsWRKYs (CsWRKY29 and CsWRKY37) conferred plants cold tolerance, and CsWRKY4/CsOCP3 (OVEREXPRESSOR OF CATIONIC PEROXIDASE 3) interacted with CsICE1 and inhibited its transcriptional activation on CsCBF1/3, demonstrating the relevance of CsCBF cascade pathway on cold tolerance of tea plants (Peng et al., 2022; Zhao et al., 2022).

The expression of *CBFs* is regulated by several types of transcriptional activators or repressors involved in light signaling, phytohormones signaling, circadian rhythms and Ca^2+^ signaling (**Figure 2**). Recent studies have shown that the PIFs (PIF3, 4, and 7), downstream genes of photoreceptor and thermosensor phyB, negatively regulates the expression of *CBF* and freezing tolerance of *Arabidopsis* (Leivar et al., 2008; Lee and Thomashow, 2012). Chilling stress initiates the formation of CBFs-PIF3-phyB complex which later serve to control the cold adaption (Jiang et al., 2020; Xu and Deng, 2020). The transcription factors (TFs) in hormone signaling maintain the homeostasis of CBF levels. For instance, the *CBFs* expression are repressed by ethylene insensitive 3 (EIN3) in ethylene pathway, but up-regulated by brassinazole-resistant 1/brassinosteroid insensitive 1-EMS-supressor 1 (BZR1/BES1) in brassinosteroids signaling (Shi et al., 2012; Li et al., 2017b). Circadian rhythms core genes are likely to antagonistically function to keep the rhythmic expression of *CBF.* Circadian clock associated 1/late elongated hypocotyl (CCA1/LHY) are shown to activate the expression of *CBFs* by binding to their promoters, while pseudo-response regulators (PRRs) inhibit the expression of *CBFs* (Nakamichi et al., 2009; Dong et al., 2011). In Arabidopsis, CCA1/LHY regulates cold-responsive *DREB1* expression only under gradual decrease in temperature during the day, whereas rapid drop in the temperature can induce the cytosolic calcium levels and activate Ca^2+^ signaling (Kidokoro et al., 2017). Ca^2+^ signaling impairment prevents *CsCBF* expression but accumulates higher catechins under cold conditions, suggesting their potential correlations in response to cold stress of tea plants (Ding et al., 2019). The *CsCAMTA2* (orthologous gene of *CAMTA3* in *Arabidopsis*) was strongly up-regulated in tea plant, and the *cis*-element [(G/A/C)CGCG(C/G/T) or (A/C)CGTGT, CsCAMTA targeted] was observed in the promoter of *CsCBF1* and *CsCBF2* (Zhou et al., 2022b). There had been at least 8 *Calmodulin-like* (*CBL*) genes and 25 *CIPK* genes identified in tea plants, which were further divided into four and five subfamilies. Of them, four *CsCBLs* (*CsCBL1/3/5/9*) and nineteen *CsCIPKs* genes were significantly induced by cold stress. Studies have shown that CsCBL1 could interact with CsCIPK1/10b/12, while CsCBL9 was found to interact with CsCIPK1/10b/12/14b; hence, the Ca^2+^-CsCBL-CsCIPK module mediated cold stress signaling in tea plant was proposed (Li et al., 2019b; Ma et al., 2019; Wang et al., 2020). In *Arabidopsis*, it was reported that CAMTAs worked together to suppress the SA (salicylic acid) synthesis by targeting *EDS1 (ENHANCED DISEASE SUSCEPTIBILITY 1)* and to improve freezing tolerance (Kim et al., 2013).

Furthermore, the post-translational modification of CBFs is important in cold tolerance. For instance, cytosolic redox protein thioredoxin h2 interacts with CBF and reduces the transformation of oxidized CBF oligomers (inactive) to active monomers, whereby this structural switching and functional activation of CBFs confers the plant with cold tolerance (Lee et al., 2021). It is worth to note that epigenetic regulation, including DNA methylation, chromatin remodeling, and small RNA regulation, also extensively influences the cold tolerance of plants throughout the entire life (Park et al., 2018). A recent study showed that hundreds of cold-responsive genes, including *CsCBF4* and *CsUGT91Q2*, were significantly demethylated during cold stress, indicating that DNA methylation is involved in cold response of tea plants (Tong et al., 2021). Besides, the histone deacetylases were also reported to participate in the cold stress response of tea plants. Low temperature reduced the transcription of *HD2 type histone deacetylase* in tea plant, indicating that chromatin remodeling mediated by histone modifications may regulate the expression of cold-responsive genes (Ma et al., 2013; Yuan et al., 2020). Degradome sequencing has identified 763 related cleavage target genes and miRNAs associated with cold stress tolerance. There were 74 and 91 differentially expressed microRNAs (miRNAs) identified from cold-tolerant ‘Yingshuang’ and cold-sensitive ‘Baiye 1’ cultivars, respectively. Of them, miR156, miR159, and miR396 showed distinct expression patterns among different cold-sensitive tea varieties under cold conditions (Zhang et al., 2014b). In addition, 14 circular RNAs have been identified to contribute to the chilling tolerance of tea plant (Huang et al., 2023).

### Identification and characterization of cold-responsive genes in tea plants

The innovation of genomic and transcriptomic sequencing, together with functional genomics, have identified a total of 128 gene/families involved in cold response in tea plants (**Table 1**). For example, the cold-responsive bZIP transcription factor *CsbZIP6* and *CsbZIP18* were experimentally evidenced to reduce the freezing tolerance of tea plants by ABA-independent and ABA-dependent pathway, respectively (Wang et al., 2017; Yao et al., 2020b). In addition, a total of 89 structural genes involving in sugar signaling, redox process, ascorbic acid metabolism, hormone signaling, carotenoid biosynthesis, terpenoid metabolism, Ca^2+^ signaling, osmoregulator, amino acid metabolism, fiber signaling, and light signaling, were also identified and characterized to be associated with cold tolerance of tea plants. Correspondingly, 59 sugar-related genes engaged in sugar metabolism, transportation and signaling are solidly stimulated, including the beta-amylase gene (*CsBAM*), disproportionating enzyme gene (*CsDPE2*), fructokinase gene (*CsFRK*), invertase gene (*CsINV5*), Suc-phosphate synthase gene (*CsSPS*) and raffinose synthase gene (*CsRS2*) (Yue et al., 2015). Sugar signaling and osmoregulator related genes regulate the cold tolerance of tea plants mainly through the osmotic-dependent pathway. Overexpression of *Invertase 5 (CsINV5)* enhanced the cold tolerance of transgenic *Arabidopsis* through up-regulating the transcription of *HXK2* and *P5CS1/2* (Qian et al., 2018). *CsSWEET1a*, *CsSWEET16* and *CsSWEET17* improved the freezing resistance of plants by promoting sugar transport across the plasma membrane (Wang et al., 2018; Yao et al., 2020a). The sequences of proline biosynthesis and degradation have been identified and available at NCBI, *CsP5CS* (pyrroline-5-carboxylate synthase, KJ143742.1), *CsOAT* (Ornithine-D-aminotransferase, KJ641844.1) and *CsP5CR* (pyrroline-5-carboxylate reductase, KY368574), *CsP5CDH* (pyrroline-5-carboxylate dehydrogenase, KY368572) and *CsProDH* (Pro-dehydrogenase, KY368573) included (Ban et al., 2017).

**Table 1:** List of the genes/gene families involved in cold tolerance of tea plant.

Category	Functional type	Gene symbol
Transcription factors	Expression analysis	*RAV, GSP, CAMTA, GARP, NLR, SAP, SDIR*
	Cloning identification	*BES1, C2H2-ZFP, CIGR, CPP, FHY3/FARI, DELA, DREB, GRF, MYB, NAC, WOX, ZF-HD, bHLH*
	Functional characterization	*bZIP, CBF, ICE, WRKY, HSF*
Structural genes	Sugar signaling	*AMY, BAM, FRK, GLU, GolS, HXK, TPP, RS, INV, UGT, SUT, TIP, TMT, SCAF, SWEET, PMI, SUS, SPS, PMM*
	Regulator genes	*AOX, C5-MTase, CIPK, CPK, CSD, dMTase, MKK, MPK, PLD, SCPL, DPE, SNRK, HDAC, MIEL, BAP, RAC*
	Redox	*CAT, GPX, GST, POD, PPO, SOD, GSHS, GGP, GME, GMP, GPP, MIOX*
	Cell remodeling	*AGP, ENODL, AXY, API, GALT, RRT, PPME, XTH, UXS, GAE, XUT, PMEI, TBL*
	Hormone signaling	*LOX, NCED, G3O2, GR, DHN, JAZ, IPT, PNPO, GalLDH, GalUR, DHAR, GalUR, AO, APX, MIOX, TTL*
	Carotenoid biosynthesis	*CHXB, CHXE, CRTISO, PDS, ZDS, PSY, Z-ISO*
	Terpenoid metabolism	*TPS, DXS, DXR, HDS, LCY, IPT*
	Ca^2+^ signaling	*CAM, CBL, CML*,
	Osmoregulator	*LEA, P5CS, AQP, FAD*
	Amino acid Metabolism	*GS, ARG*
	Fiber signaling	*HCT, CesA*
	Light signaling	*psbA, psbD*

Please check **Supplemental Table 1** for details.

Besides, the phenylpropanoid pathway serves as a rich source of metabolites in plants, as a starting point for the biosynthesis of lignin, flavonoids and coumarins (Fraser and Chapple, 2011; Hori et al., 2020; Oliveira et al., 2020). Recent studies showed upregulation of lignin biosynthesis genes along with downregulation in cellulose biosynthesis genes under osmotic stresses in tree species (Wildhagen et al., 2018; Chen et al., 2019; Hori et al., 2020). Additionally, an increased level in xyloglucan endotransglucosylase/hydrolase (XTH) and expanding proteins, affecting the cell wall plasticity and reinforcement of the secondary wall with hemicellulose and lignin deposition to increase cell wall thickening were highlighted (Gall et al., 2015). In accordance with these data, our RNAseq data revealed many upregulated genes related to the cell wall remodeling and biosynthesis in tea plant (*UDP-Arap*, *XTH30*, *AGPS1*, *BGLU*, *ENODL2*, *AXY4*, *UEL-*1, *PRP-F1*, *API*, *PPME*, *GALT6*, *GATL7*, *UXS2*, *UXS4*, *TBL32*, *GlcAT14A*, *XUT1*, *GAE3*, *4CL*, *API*, *RRT1*, *rfbC*, glucan endo-1,3-beta-glucosidase 7-like and 8-like, etc.) confirming the importance of this pathways in the long-term cold stress in tea plant. Additionally, elevated expression of beta-glucosidase (BGLU) that catalyzes intermediates for cell wall lignification synthesis was observed in tea. Also, several DEGs (*RRT1*, *PPME*, *XTH*, *UXS2*, *UXS4*, *GAE3*, *XUT1*) related to xyloglucan and pectin biosynthesis were upregulated in tea leaves under the long-term cold stress. Among them, *RRT1* (*RG-I RHAMNOSYLTRANSFERASE 1*) is required for both cellular adhesion and cell wall plasticity (Takenaka et al., 2018). *PPME*, *pectinesterase-like PMEs* (*PECTIN METHYLESTERASE INHIBITORs*) maintains apoplastic Ca^2+^-homeostasis, controlling stomatal movements and in regulating the flexibility of the guard cell wall (Wu et al., 2018). Previous studies have also suggested that inhibiting the pectin methylesterase activity of tea plants, including *Pectin Methylesterase Inhibitor* 2 and 4 (*CsPMEI2* and *CsPMEI4*), slightly reduces the cold tolerance of transgenic *Arabidopsis* (Li et al., 2021). XTHs (Xyloglucan endotransglucosylase/hydrolase) cuts and re-joins hemicellulose chains in Plant cell wall, contributing to wall assembly, affecting cellulose deposition (Wu et al., 2018). Additionally, more genes related to pectin biosynthesis were found upregulated under long-term cold stress in tea plant, namely *UXS2/4* (*UDP-GLUCURONIC ACID DECARBOXYLASE 2/4*) and *GAE3* (UDP-D-GLUCURONATE 4-EPIMERASE 3). These genes are required for the biosynthesis of heteroxylans and xyloglucans and for the side chains of pectin (Kuang et al., 2016; Borg et al., 2021).

Cell walls remodeling proteins contain hydroxyproline-rich O-glycoproteins (HRGPs), which is classified into extensins (EXTs), arabinogalactan-proteins (AGPs) and Hyp/Pro-rich proteins (H/PRPs) (Cassab and Varner, 1988; Basu et al., 2015; Ajayi et al., 2021). According to our results, a set of genes involved in H/PRPs and AGPs metabolism (*AGPS1*, *UEL-1*, *API*, *GALT6*, *GATL7*, *GlcAT14A*, *ENODL2*, *PRP-F1*, etc.) were highly upregulated in tea plant suggesting that glycosylation of HRGPs is an important responsive mechanism under the long-term stress. Additionally, some genes (e.g., *TBL27/32*) related to O-acetylation of polysaccharides were upregulated under long-term cold in tea plant which is consistent with some earlier findings (Sun et al., 2020). O-Acetylation of polysaccharides change the physicochemical properties and acetyl-substituents inhibit the enzymatic degradation of wall polymers (Gall et al., 2015) suggesting the important role of the both processes for the long-term stress responses of tea plant. Thus, the increasing the cell wall plasticity, thickness and hydrophobicity by lignin biosynthesis, glycosylation of HRGPs, o-acetylation of polysaccharides, pectin biosynthesis and branching, xyloglucan and arabinogalactan biosynthesis can serve as important mechanisms of long-term cold responses in tea plant.

A well-known effect of abiotic stress in plants is the production of ROS, which can eventually oxidize lipids, proteins, and DNA, and thereby trigger the cell death (Akula and Ravishankar, 2011; Bartwal et al., 2013; Estravis-Barcala et al., 2020). Redox process and ABA metabolism regulated cold tolerance mainly through scavenging reactive oxygen species. For example, *Glycosyltransferase CsUGT91Q2*, *CsUGT78A14*, and *CsUGT71A59* confer cold resistance to tea plant by improving the ROS clearance ability (Zhao et al., 2019a; Zhao et al., 2019b; Zhao et al., 2021). According to our recent results, in tea plant several upregulated DEGs related to lipid metabolism were upregulated under long term cold stress. For examples, the homologs of *SEC14*, an important regulators of phospholipid metabolism (Campos and Schaaf, 2017), *EDR2*, a negative regulator of cell death (Vorwerk et al., 2007), and genes encoding remorin-like (REMs) proteins accumulated in lipid rafts and physically interact with receptor-like kinases (Cai et al., 2020), probable phospholipid hydroperoxide glutathione peroxidase (*PHGPX*) participates in scavenging of lipid hydroperoxide (Jain and Bhatla, 2014), endoplasmic reticulum oxidoreductin-1-like (*ERO1*) participating in protein folding under oxidative stress (Matsusaki et al., 2019), probable carboxylesterase 11 (*CXE11*) which is involved in the catabolism of volatile esters such as butyl and hexyl acetate and activation of MeJA signaling (Cao et al., 2019), luminal-binding protein genes (*BIP5*-like) which increase in anti-oxidative defenses under water stress in transgenic tabacco and soybean (Valente et al., 2009). These results suggest the lipid stabilization against ROS can be an important mechanism of the long-term cold and freezing responses in tea plant.

Similarly, cold-induced enzyme or hormone pathway genes also affect the cold tolerance of tea plants. According to the recent studies, hormone-signaling pathways are consistently up-regulated under cold stress, which are involving in those of JA, brassinosteroids (BRs), and ABA (Wisniewski et al., 2014; Zheng et al., 2022). Interestingly, auxin signal transduction is activated in the opposite pattern with ethylene transduction it some tree species (Estravis-Barcala et al., 2020). In tea plant several new upregulated DEGs involved in hormone signaling were upregulated under the long-term cold stress (*GID1C*-like, *LOG3*-like, *ILR1-like6*, *TTL1*, *TTL3*, and *2g29380*). These genes are related to the abovementioned signaling pathways. For example, *GID1* (*GA INSENSITIVE DWARF1*) can bind negative regulators of GA responses called DELLA proteins (Hauvermale et al., 2014). LOG is a cytokinin-activating enzyme plays a pivotal role in regulating cytokinin activity (Kuroha et al., 2009). *ILR1* (*IAA-LEUCINE RESISTANT 1*) regulates the rates of amido-IAA hydrolysis resulting in activation of auxin signaling (Sanchez Carranza et al., 2016). *TTL1* (*TETRATRICOPEPTIDE-REPEAT THIOREDOXIN-LIKE 1*) regulates the transcript levels of several dehydration-responsive genes, such as *CBF2*, *ERD1* (early response to dehydration 1), *ERD3*, and *COR15a* (Rosado et al., 2006; Lakhssassi et al., 2012). These results indicate a complex transcriptional landscape in response to abiotic stress, and in particular they show highly variable interactions between different hormone signal transduction pathways.

Long-term cold stress down-regulated *CsLOX* expression while short period of low temperatures induced the expression of *CsLOX1*, 6 and 7, which highlights the role of JA in triggering and regulating cold tolerance of tea plants (Zhu et al., 2018). E3 ligase gene *MIEL1* inhibited the accumulation of anthocyanin in apple by degrading MdMYB1 protein (An et al., 2017). Similar to the function of *MdMIEL1*, overexpression of the *CsIEL1* gene in *Arabidopsis* decreased anthocyanin level during cold stress, which is possibly caused by the degradation of positive regulator through 26S-proteasome-mediated ubiquitination pathway (Xing et al., 2021).

In addition, transcriptome analysis shows only 12% of cold-responsive genes are dependent on the CBF regulons in *Arabidopsis thaliana*, indicating the presence of the other low-temperature regulation pathways (Fowler and Thomashow, 2002). Indeed, several previous studies have showed that many cold-inducible genes, including *Alpha-tubulin (CaTUA), dehydrin (CsDHN1, 2), spermine synthase (CsSPMS), fatty acid desaturase (CsSAD), H1 histone (CsHis), CsbZIP* and *CsHSF* function in cold response of tea plants in a CBF-independent pathway (Paul et al., 2012; Paul and Kumar, 2013; Wang et al., 2014; Zhu et al., 2015; Ding et al., 2016). Many phytohormone (auxin, cytokinins, ABA, gibberellins, JA, ethylene and brassinosteroids) responsive genes are intimately linked to the CBF-independent regulon under cold acclimation (Zhao et al., 2014; Joshi et al., 2016; Wani et al., 2016). With the development of transcriptomics and genomics, many CBF-independent transcriptional regulation factors involved in cold adaptions would be identified.

### Exogenous feeding to improve cold tolerance in tea plants

At present, the primary method of reducing cold stress in tea production is to breed cold-resistant tea plants and optimize cultivation conditions, most likely in a greenhouse or with a protective film. Applying exogenous substances, on the other hand, are the simplest, most convenient, and most effective method (Zhang et al., 2022b). In general, 93 metabolites changed significantly under cold stress, such as catechin, flavonoid, ABA and JA (Hao et al., 2018). These results corresponded to those in which low temperature increased accumulation of flavan-3-ols and proanthocyanidins, indicating that phytohormones and secondary metabolites may contribute to cold regulation in tea plants (Zhang et al., 2014a). Indeed, treatment with plant growth regulators spermidine (0.025 mM, 0.05 mM, 0.1 mM) alleviates damages caused by cold stress in pollen tubes of tea varieties (Cetinbas-Genc et al., 2020). Exogenous application of ABA not only induces *CsCOR1* expression but also rapidly close stomata to reduce water loss, thereby ABA effectively alleviates chilling damage to plant, consistent with the changes in proline content (Li et al., 2010a; Hong et al., 2017). As efficient elicitor, exogenous methyl jasmonate application induces the expression of *CsMYBs*, and thus effectively promotes ROS scavenging and anthocyanin biosynthesis to alleviate cold stress damage (Han et al., 2022). Melatonin treatment alleviates cold stress on tea plant by improving biosynthesis antioxidant enzyme and antioxidant defense and redox homeostasis (Li et al., 2018a; Li et al., 2018b; Li et al., 2019a). Additionally, supplementation of γ-aminobutyric acid also contributes to the improvement of cold tolerance of tea plants, as exogenous application of CaCl_2,_ which has the same effect on cold tolerance. (Huang et al., 2015; Zhu et al., 2019). Airborne nerolidol and nerolidol glucoside exposure were also found to enhance cold stress tolerance of the tea plant through accumulating *CsCBF1* and *CsUGT91Q2* expression (Zhao et al., 2019b). However, the mechanism by which exogenous substances orchestrate cold tolerance has yet to be determined.

## Conclusions and perspectives

Tea is a perennial and evergreen woody crop, which is mainly cultivated in tropical and temperate regions. Low temperature stress poses serious threat to the tea plant growth and distribution. Therefore, it is critical to elucidate the physiological and molecular mechanisms through which tea plant cope with cold stress and introduce the most effective and preventive measures for cold stress. In the last few decades, the research on cold stress has mainly focused on the physiological and biochemical changes and gene expression profiles of different tea varieties during low temperature. Here, we described the putative cold sensors and signaling transduction pathways, coupled with existing research. Despite significant efforts, only a few potential cold sensors have been identified due to redundancy in sensor coding genes and challenging experimental techniques. (Zhu, 2016). To survive in adverse cold conditions, tea plant has evolved precise adaptive mechanisms, particularly known as ICE-CBF-COR pathway. Growing reports suggest the crosstalk between other factors, such as hormones, light and circadian clock pathway, and cold signaling can effectively balance the cold tolerance and plant growth, which is worthy for further research (Achard et al., 2008; Hong et al., 2017; Janda et al., 2021). Although emerging evidence shows that the transcriptional regulation, epigenetic regulation and post-transcriptional modifications played a significant role in CBF signaling, the related regulatory networks still wait for further study in tea plant. We also carefully checked the literature and listed hundreds of gene families involving in cold stress. Finally, the influence of exogenous application on tea plant was also outlined, albeit with an unclear molecular basis.

The recent comparative genomics, transcriptomics, and proteomics-based analysis have revealed large numbers of genes related to low temperature and enriched the gene resource of tea plant, expanding comprehensive understanding about process involving cold stress (Wang et al., 2013; Hu et al., 2013b; Li et al., 2019; Xia et al., 2020; Lei et al., 2021). However, the biological nature of tea plants precludes the biotechnological strategies in itself; for instance, perennial woody and self-incompatible characteristics, successful genetic transformation systems and some experimental protocols successfully used for *Arabidopsis* and other model plants cannot be fully applied in case of tea plants. Thus, the discovery of novel genes and most reliable functional identification of candidate genes is still an important but challenging topic for tea researchers.

The cold signaling, light and hormone signaling are tightly connected under cold stress. The light receptor also functions as cold sensor, suggesting the integration between temperature and photoreceptors, while the exact mechanism of cold perception needs further study. Due to resource limitations, plants tend to transfer more resources at the expense of normal growth and development to activate the defense system under cold stress, a response termed tradeoff between growth and defense. Low temperature induced the increase of growth inhibiting hormones such as ABA and JA, and the decrease of growth promoting hormone levels such as IAA and GA; therefore, unraveling the connection between hormone and cold signals is an important step for researching plant growth and development.

In the near future, establishing high-efficient transgenic system for tea plant are necessary. The rich polyphenols directly kill *Agrobacterium* as antibacterial agent and block the T-DNA transport channel to tea plant cells as protein precipitation agent, thus leading to low conversion efficiency. Thus, the co-domestication of *Agrobacterium* and issues of tea pant could be a good method to establish an efficient genetic transformation system. Compared with many other crops, tea plants need more measures to be taken to accelerate molecular design breeding for highly cold-tolerant tea plants.

## Author contributions

EX designed the project. YW collected and analyzed the data. YW and LS wrote the paper. EX, WT, LS, AM, and WT revised the paper with inputs from all authors. All authors contributed to the article and approved the submitted version.
